# Public Interest and Behavior Change in the United States Regarding Colorectal Cancer Following the Death of Chadwick Boseman: Infodemiology Investigation of Internet Search Trends Nationally and in At-Risk Areas

**DOI:** 10.2196/29387

**Published:** 2021-08-26

**Authors:** Nicholas B Sajjadi, Kaylea Feldman, Samuel Shepard, Arjun K Reddy, Trevor Torgerson, Micah Hartwell, Matt Vassar

**Affiliations:** 1 Office of Medical Student Research College of Osteopathic Medicine Oklahoma State University Center for Health Sciences Tulsa, OK United States; 2 Department of Psychiatry and Behavioral Sciences College of Osteopathic Medicine Oklahoma State University Center for Health Sciences Tulsa, OK United States

**Keywords:** Google Trends, colerectal cancer, search analytics, public health, data analytics, Chadwick Boseman, Twitter, infodemiology

## Abstract

**Background:**

Colorectal cancer (CRC) has the third highest cancer mortality rate in the United States. Enhanced screening has reduced mortality rates; however, certain populations remain at high risk, notably African Americans. Raising awareness among at-risk populations may lead to improved CRC outcomes. The influence of celebrity death and illness is an important driver of public awareness. As such, the death of actor Chadwick Boseman from CRC may have influenced CRC awareness.

**Objective:**

We sought to assess the influence of Chadwick Boseman’s death on public interest in CRC in the United States, evidenced by internet searches, website traffic, and donations to prominent cancer organizations.

**Methods:**

We used an auto-regressive integrated moving average model to forecast Google searching trends for the topic “Colorectal cancer” in the United States. We performed bivariate and multivariable regressions on state-wise CRC incidence rate and percent Black population. We obtained data from the American Cancer Society (ACS) and the Colon Cancer Foundation (CCF) for information regarding changes in website traffic and donations.

**Results:**

The expected national relative search volume (RSV) for colorectal cancer was 2.71 (95% CI 1.76-3.66), reflecting a 3590% (95% CI 2632%-5582%) increase compared to the expected values. With multivariable regression, the statewise RSV increased for each percent Black population by 1.09 (SE 0.18, *P*<.001), with 42% of the variance explained (*P*<.001). The American Cancer Society reported a 58,000% increase in CRC-related website traffic the weekend following Chadwick Boseman’s death compared to the weekend before. The Colon Cancer Foundation reported a 331% increase in donations and a 144% increase in revenue in the month following Boseman’s death compared to the month prior.

**Conclusions:**

Our results suggest that Chadwick Boseman’s death was associated with substantial increases in awareness of CRC. Increased awareness of CRC may support earlier detection and better prognoses.

## Introduction

Colorectal cancer (CRC) currently has the third highest mortality rate among cancers in the United States [1]. Implementing enhanced screening, namely by colonoscopy, has led to a significant decline in CRC mortality rates in the older population; [2] however, some populations remain at disproportionately high risk. African Americans have the highest incidence and mortality rates for CRC of any ethnic group; however, they are less likely to receive appropriate screening [3]. Additionally, in 2020, Rogers et al [4] identified regions in the United States that are associated with higher rates of early onset CRC, specifically among African American males, highlighting the need to focus on improving outcomes in this population. Raising awareness of CRC disparity among stakeholders and those at increased risk is a necessary component for achieving improved outcomes.

Infodemiology is the scientific study of distributions, determinants, and characteristics of information in an electronic medium, specifically the internet, or in a population, with the ultimate goal of informing public health and public policy [5]. Infodemiologic frameworks have provided a methodological means of analyzing public interest and awareness of medical conditions [6]. A few established infodemiologic metrics include aggregated data sets revealing patterns of information-seeking or information utility on websites and social media [7], the discourse and discussion found in web-based forums or blogs, and a population’s activities on search engines over time [5,8]. Using infodemiologic metrics for medical research provides a real-time data stream reflecting the dynamics of information prevalence and utility that may be difficult to capture with traditional methodologies. Using infodemiologic methods may allow researchers to gauge the public interest in and awareness of CRC in at-risk populations.

As efforts are needed to raise CRC awareness, the entertainment industry may be well positioned to exert a positive influence on public awareness. The role of celebrity influence in health communications has been well studied by communications researchers, and communications scholarship is a useful source for understanding web-based searching and engagement behaviors related to publicized celebrity health information [9,10]. The influence of celebrity health on the public’s awareness of medical conditions has been expanded upon by medical infodemiology research. In one infodemiologic study, media coverage of public figures disclosing a cancer diagnosis was shown to generate substantial public interest in various types of cancer. For example, large spikes in internet searches for *pancreatic cancer* were associated with actor Patrick Swayze’s public announcement of his pancreatic cancer diagnosis. Similar spikes were observed when Steve Jobs took medical leave from Apple after being diagnosed with pancreatic cancer. Additional large spikes in searches for *pancreatic cancer* were observed following both of their deaths. In some cases, this coverage led to greater measurable increases in awareness than that of traditional awareness campaigns [11]. Although the study by Kaleem et al [11] examined numerous spikes in interest for many cancers, it did not show isolated peaks for CRC due to paucity of public figures announcing they had colon cancer. The influence of celebrity illness and death from CRC may have important implications for increasing awareness of CRC.

On August 28, 2020, Chadwick Boseman, star of the Marvel movie *Black Panther*, died at age 43 of colorectal cancer [12]. Boseman’s role in this film helped to normalize African heritage and culture in the United States and is considered to have significant cultural importance in the African American community [13-15]. As Boseman was a prominent figure in the United States and in the African American community, his death presents a unique opportunity to study the influence prominent public figures have on public awareness and behaviors concerning CRC in the United States. Thus, we examined internet searching data before and after Boseman’s death to examine the potential influence on public interest regarding CRC, nationally and in states containing regions at high risk for CRC. Additionally, we contacted the American Cancer Society (ACS) and the Colon Cancer Foundation (CCF) to inquire about differences in website traffic and donation revenue around the time Boseman’s death was disclosed to the public. Findings from this study may strengthen our understanding of the influence that public figures have on public health and may help to raise awareness of CRC among at-risk communities.

## Methods

### Data Sources

Three data sources were used for gauging public interest in CRC surrounding Boseman’s death: Google Trends (GT), Wikipedia, and Twitter. We used Google Trends [16] because it is useful for identifying regional population interests and behaviors regarding medical information [8]. GT is an open database that presents population-based Google search trends over specified time periods, allowing for nearly real-time observation. GT reports regional search volumes for selected topics over time. The relative search volume (RSV) for a topic or search term is represented as a value ranging from 0 to 100, with 100 indicating peak popularity during the designated time frame. As such, GT data may serve as a proxy for the relative interest in or awareness of a given topic in a specific region over a specific time [11]. GT data is a particularly useful tool in the field of oncology, as internet searching for cancer-related topics over time has been shown to significantly correspond with the statewise incidence and mortality rates of certain cancers in the United States, including CRC [17]. On September 13, 2020, we collected national RSV data for the topic *colorectal cancer* in the United States. We used the date range of June 13 through September 11, 2020, to observe trends prior to, during, and shortly after Boseman’s death, aiming to minimize confounding from any related events.

Additionally, we collected statewise RSV data for the topic *colorectal cancer* in the United States during the peak interest time. The peak interest time was designated as 1 week before Boseman’s death to 1 week after (August 21 through September 4, 2020) to capture the immediate public response associated with Chadwick Boseman’s death. Data were collected on September 13, 2020, under the “subregion” category for the United States. Each state’s RSV was then paired with its most recently reported CRC incidence rate [18] as well as its percent Black population [19]. Pairing these data allowed us to explore associations between a state’s RSV for *colorectal cancer* during peak interest with its CRC incidence rate and its percent Black population.

Wikipedia is the most frequently used source for seeking medical information on the internet, and it is therefore a valuable source for assessing public interest in medical topics [20]. We used the Pageviews Analysis [21] tool to acquire the number of visits to the Wikipedia pages for “Colorectal cancer” and “Chadwick Boseman” over the same time period. Pageviews Analysis was used by Brigo et al [22] to provide evidence of associations with celebrity appearances and increased Wikipedia searching that suggested increased public knowledge of multiple sclerosis. Twitter is also a useful source for infodemiologic studies, and it has been used to assess the impact of awareness campaigns and analyze public engagement with medical information [23-26]. Sprout Social [27] was used to acquire the number of tweets containing the text “colon cancer” and “chadwick boseman”. We selected similar terms for Wikipedia and Twitter data to ensure content uniformity across platforms. The temporal trends for Wikipedia and Twitter were worldwide trends and were observed over the same time period as the US national GT data.

### Analyses

With the national GT data, we used an autoregressive integrated moving average [28] (ARIMA) model to forecast the expected relative search volume for *colorectal cancer* had Boseman’s death from CRC not been publicly disclosed, comparing expected values to observed values. Using the statewise RSV data from the peak interest time, we employed bivariate regression models on the statewise CRC incidence rate and on the percent Black population. We then used multivariable models considering both parameters. We performed statewise regression analysis on all 50 states and the District of Columbia. We then performed a subanalysis on the 19 states shown by Rogers et al [4] to contain so-called CRC hot spots—regions with disproportionately high rates of CRC among African Americans, particularly young African American males.

To explore the behavior change associated with Boseman’s death, we obtained data from the ACS regarding the percent increase in colon cancer–related traffic on their website the weekend following Boseman’s death compared to the weekend prior. We also obtained the number of daily donations received by the CCF and the percent increase in revenue seen 1 month following Boseman’s death compared to the month prior. Additionally, both organizations provided estimates of percent increases in total revenue 1 month following Boseman’s death compared to the same time period in 2019.

All statistical analyses were performed using R, version 4.0.2 (R Foundation for Statistical Computing) [29]. Statistical significance was defined as *P*<.05. The Oklahoma State University Center for Health Science Institutional Review Board determined that this project did not qualify as human subject research as defined in 45 CFR 46.102(d) and (f); therefore, it was not subject to further oversight.

## Results

**Figure 1:**
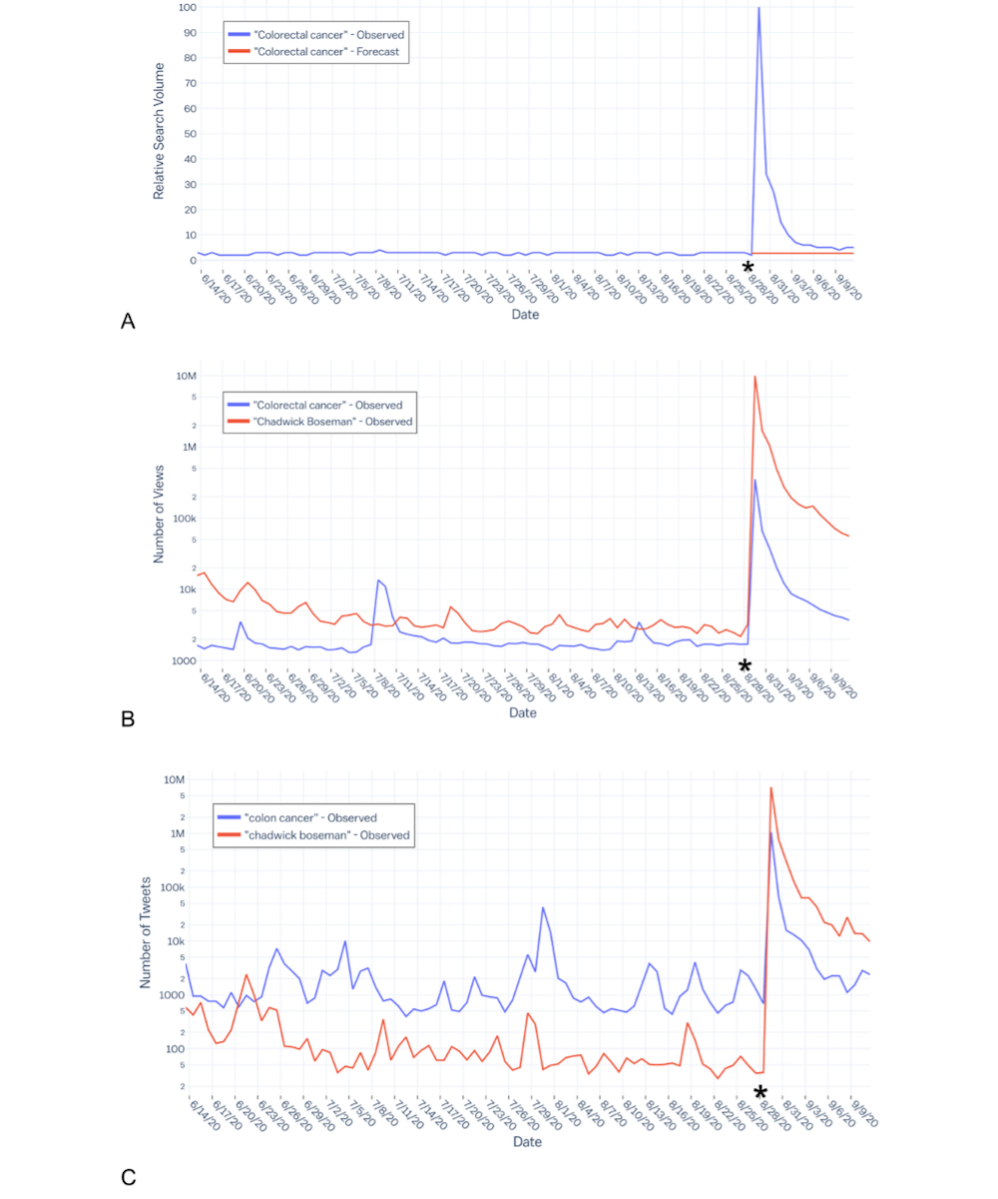
(A) Relative search volumes for *colorectal cancer* in the United States before and after Chadwick Boseman’s death on August 28, 2020 (indicated by the asterisk). The expected forecast from the autoregressive integrated moving average model is shown by the red line. (B) The total number of visits (worldwide) to the “Colorectal cancer” and “Chadwick Boseman” Wikipedia pages before and after Chadwick Boseman’s death. The vertical axis is on a logarithmic scale, with each large tick mark representing an order of magnitude. (C) The number of tweets containing the text “colon cancer” or “chadwick boseman”. The vertical axis is on a logarithmic scale.

The auto.arima function in R established parameters for the ARIMA model to forecast values based on the historical mean RSV. The expected national RSV for *colorectal cancer* was 2.71 (95% CI 1.76-3.66). The observed peak RSV (100) occurring on August 29, 2020, reflects a 3590% (95% CI 2632%-5582%) increase in national RSV compared to expected values. Large spikes in Wikipedia searches and Twitter keywords were observed during the peak interest time. The GT forecast comparison and temporal trends for Wikipedia and Twitter can be found in Figure 1.

The statewise bivariate regression models for all 50 states and the District of Columbia showed that for each 1 point increase in incidence rate, the RSV would decrease by 0.25 (SE=0.4, *P*=.53), a nonsignificant finding; however, for each 1% increase in the Black population, the RSV increased by 1.06 (SE=0.18, *P*<.001). With the multivariable model, the statewise RSV further decreased per point of incidence to –0.47 (SE=0.3, *P*=.13) and increased for each percent Black population to 1.09 (SE=0.18, *P*<.001), with 42% of variance explained by the model (*P*<.001). The coefficients in the subanalysis adjusted model for the 19 states containing hot spot regions were in the same direction, but to a lesser degree, and they accounted for 33% of the variability in RSV (*P*=.01). Full results of the regression models are displayed in Table 1.

The American Cancer Society reported a 58,000% increase in colon cancer–related website traffic the weekend following Boseman’s death compared to the weekend before. The CCF reported receiving 595 donations from July 29 to August 28, 2020, and 2565 donations from August 28 to September 30, 2020, representing a 331% increase in the number of donations the month following Boseman’s death. The ACS reported a 35.4% increase in total revenue the week following Boseman’s death compared to the same time in 2019, and the CCF reported a nearly 500% increase in total revenue the week following Boseman’s death compared to the same time period in 2019. Additionally, the CCF reported a 144% increase in total revenue the month following Boseman’s death compared to the month prior, and they stated that they anticipated additional daily revenue influx as companies continued to run campaigns on the CCF’s behalf following Boseman’s death.

**Table 1:** Correlation of relative search volume (RSV) with colorectal cancer (CRC) incidence and percent Black population.

		All 50 US states and DC^a^					CRC hot spot states^b^ (n=19)			
		Coefficient	SE	*P* value	*R* ^2^	Coefficient		SE	*P* value	*R* ^2^
**Bivariate model**										
	Incidence of CRC	–0.25	0.40	.53	<.01	–1.33		0.39	*.003* ^c^	0.38
	Percent Black population	1.06	0.18	*<.001*	0.40	0.63		0.28	*.04*	0.18
**Multivariable model**										
	Incidence of CRC	–0.47	0.30	0.132	0.42	–0.56		0.32	.10	0.33
	Percent Black population	1.09	0.18	*<.001*	0.42	0.75		0.24	*.007*	0.33

^a^DC: District of Columbia.

^b^States with hot spot counties: Alabama, Arkansas, Florida, Georgia, Illinois, Indiana, Kentucky, Louisiana, Maryland, Mississippi, North Carolina, Ohio, Oklahoma, South Carolina, Tennessee, Texas, Virginia, West Virginia.

^c^Italic text indicates statistical significance.

## Discussion

### Principal Findings

**Figure 2:**
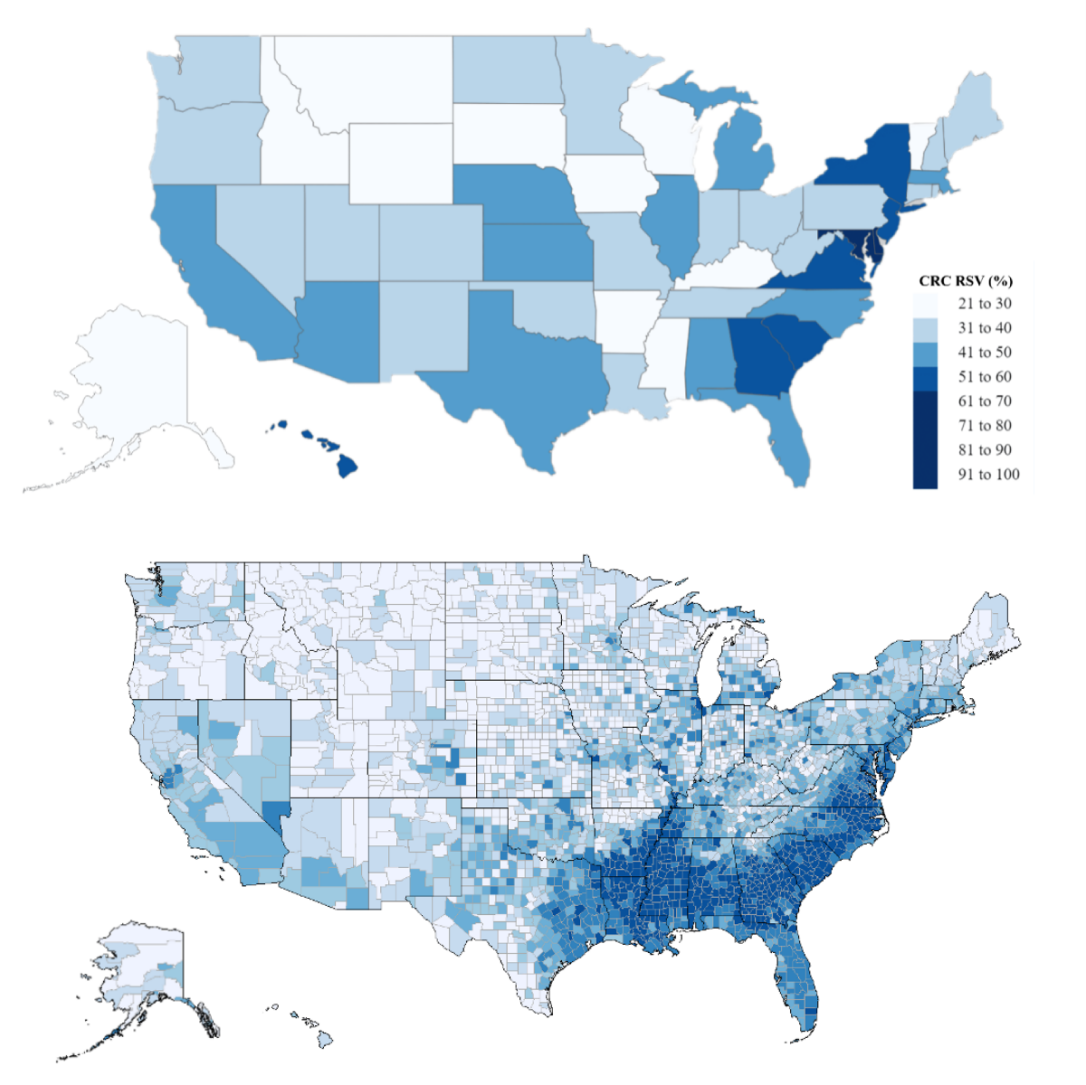
(Top) RSV on Google Trends for the term *colorectal cancer* during peak interest surrounding the death of Chadwick Boseman. (Bottom) Percentage of Americans who identified as Black/African American on the 2015 American Community Survey [33]. CRC: colorectal cancer; RSV: relative search volume.

Our results suggest that Chadwick Boseman’s death was associated with substantial increases in the national interest in and awareness of CRC in the United States. Although a state’s RSV for *colorectal cancer* during the peak interest time was not statistically associated with its CRC incidence rate, our results suggest that the RSV for *colorectal cancer* during peak interest time was significantly associated with a state’s percentage of Black residents. Significant increases in searches for CRC-related topics in states with higher percentages of African American residents suggest that Boseman’s death effectively increased awareness among at-risk populations. This is an important finding given that Rogers et al [4] found young (ages 20-49) non-Hispanic Black men to have the lowest early onset CRC survival rates among all ethnic groups in 232 hot spot counties—one of which is Anderson County, South Carolina, Boseman’s birthplace and home county. Numerous studies have shown that raising awareness of factors for heightened risk of CRC corresponds with increased willingness to undergo CRC screening procedures and improved attitudes toward CRC [30-32]. Increased interest in and awareness of CRC among at-risk populations, namely African Americans, may lead to earlier detections and better prognoses. The RSVs for *colorectal cancer* can be visualized in Figure 2. For comparison, we have included a US map showing the African American population density by state population for comparison [33].

The spikes in Wikipedia searches for *colorectal cancer* and in tweets containing “colorectal cancer” coinciding with Boseman’s death were the all-time largest recorded volumes for their respective platforms, putting into perspective the massive magnitude of increased searching for CRC topics following Boseman’s death. Boseman’s death was announced on his personal Twitter account shortly after his death from CRC, and the announcement is now the single most “liked” tweet in Twitter history [34]. Our findings support the use of Wikipedia and Twitter data as reliable indicators of public interest resulting from public disclosure of celebrity illness and necessitate further exploration of these platforms as research tools.

We found substantial increases in website traffic and donations to two prominent cancer organizations following Boseman’s death, suggesting increased public financial support of CRC research and awareness campaigns. Although these findings represent website traffic and donation behavior over a short period of time, they are compelling and promising. Chadwick Boseman’s death may serve as a catalyst toward increasing public awareness of CRC, leading to increased financial support for CRC research and awareness campaigns. Increasing CRC research funding is necessary, as it is disproportionately underfunded [35]. In 2015, CRC caused 50,620 deaths and received US $18 million in funding from non-profits, whereas breast cancer caused 41,070 deaths and received US $460 million [36]. Funding from the National Cancer Institute was similarly distributed [35,37]. Long-term improvements in health outcomes are best supported by policy, evidence-based medicine, and public health initiatives; however, events such as Boseman’s death have the potential to overcome cultural and societal barriers to positive health behavior in ways that the aforementioned factors cannot.

As mentioned previously, communications research [9,10] and infodemiologic GT studies have demonstrated the significant impact public figures can have on the public’s awareness of various medical conditions. Importantly, we assert that *increased awareness not resulting in measurable positive behavior change* is, while still admirable and necessary, only a partial victory. Until recently, the impact of a public figure’s disclosure of illness regarding CRC was unknown. In line with our own findings, one recently published study by Naik et al [38] examined internet search interests in CRC-related topics following Chadwick Boseman’s death and found increases in searches on Google and Wikipedia comparable to our own. Moreover, the study found proportionally higher increases among areas with higher proportions of Black Americans, a novel and important finding that should be used to increase CRC education among this at-risk community. Further, novel components within our study include additional infodemiologic parameters (Twitter) to measure increased interest and awareness, showing significant increases in activity and the inclusion of data from the ACS and the CCF demonstrating increased donations following Boseman’s death. Our study complements that by Naik et al [38] and suggests positive behavior changes regarding CRC following Boseman’s death. Together, these findings contribute to the international literature base by providing evidence of positive associated behavior change that goes beyond increased internet searching, awareness, or interest following the death of the actor. Further research regarding the direct impact of Chadwick Boseman’s death on CRC screenings and attitudes toward CRC screening among African Americans is warranted, as it cannot be assessed herein.

### Strengths and Limitations

Our study has several strengths. To our knowledge, our study is the first of its kind to provide evidence of positive behavior change in the form of donations associated with Chadwick Boseman’s death. Our methodology was adapted from published literature that used GT and ARIMA models to provide evidence for increased awareness of suicide prevention resources following a nationally publicized event [39]. The data provided by the ACS and CCF reflect increases in public interest and support for CRC research, possibly related to Boseman’s death, and these data are unique to our study. Limitations of this study include the limited time frame for observing RSV, website traffic and donations, use of few search terms, and lack of access to donation data entailing actual dollar amounts. The limited observation period limits the generalizability of our findings to the time periods in which we searched, and the long-term influence of Boseman’s death cannot be ascertained from our findings. It is also not possible to make causal claims based on our data, and our results should be interpreted accordingly. Other events could have influenced our outcomes; thus, the potential for confounding factors is present.

### Conclusion

National public interest in colorectal cancer substantially increased relative to predicted values following Chadwick Boseman's death. A state’s RSV for *colorectal cancer* immediately surrounding Boseman’s death was significantly associated with its percentage of Black residents, possibly suggesting increased CRC awareness among this population. Increased interest among at-risk populations associated with Chadwick Boseman’s death may lead to improved health outcomes and attitudes regarding CRC. Website traffic and revenue to prominent cancer organizations increased following Chadwick Boseman’s death. Increased public awareness of CRC associated with Chadwick Boseman’s death may lead to increased support for CRC research and awareness campaigns.

## Abbreviations

ACS: American Cancer Society
ARIMA: autoregressive integrated moving average
CCF: Colon Cancer Foundation
CRC: colorectal cancer
GT: Google Trends
RSV: relative search volume
